# Intracranial pressure waveform characteristics in idiopathic normal pressure hydrocephalus and late-onset idiopathic aqueductal stenosis

**DOI:** 10.1186/s12987-021-00259-y

**Published:** 2021-05-26

**Authors:** Lauren M. Green, Thomas Wallis, Martin U. Schuhmann, Matthias Jaeger

**Affiliations:** 1 Department of Neurosurgery, Wollongong Hospital, Loftus Street, Wollongong, NSW 2500 Australia; 2 Department of Neurosurgery, Eberhard Karls University Hospital, Tübingen, Germany; 3 Illawarra Health and Medical Research Institute, Wollongong, NSW Australia; 4 University of New South Wales, South Western Sydney Clinical School, Liverpool, NSW Australia; 5 University of Wollongong, Wollongong, NSW Australia

**Keywords:** Idiopathic normal pressure hydrocephalus, Aqueductal stenosis, Intracranial pressure, Neuromonitoring, Intracranial compliance, Intracranial pulsations

## Abstract

**Background:**

Idiopathic normal pressure hydrocephalus (iNPH) and late-onset idiopathic aqueductal stenosis (LIAS) are two forms of chronic adult hydrocephalus of different aetiology. We analysed overnight intracranial pressure (ICP) monitoring to elucidate ICP waveform changes characteristic for iNPH and LIAS to better understand pathophysiological processes of both diseases.

**Methods:**

98 patients with iNPH and 14 patients with LIAS from two neurosurgical centres were included. All patients underwent diagnostic overnight computerised ICP monitoring with calculation of mean ICP, ICP heartbeat related pulse wave amplitude calculated in the frequency domain (AMP) and the time domain (MWA), index of cerebrospinal compensatory reserve (RAP) and power of slow vasogenic waves (SLOW).

**Results:**

ICP was higher in LIAS than iNPH patients (9.3 ± 3.0 mmHg versus 5.4 ± 4.2 mmHg, p = 0.001). AMP and MWA were higher in iNPH versus LIAS (2.36 ± 0.91 mmHg versus 1.81 ± 0.59 mmHg for AMP, p = 0.012; 6.0 ± 2.0 mmHg versus 4.9 ± 1.2 mmHg for MWA, p = 0.049). RAP and SLOW indicated impaired reserve capacity and compliance in both diseases, but did not differ between groups. INPH patients were older than LIAS patients (77 ± 6 years versus 54 ± 14 years, p < 0.001).

**Conclusions:**

ICP is higher in LIAS than in iNPH patients, likely due to the chronically obstructed CSF flow through the aqueduct, but still in a range considered normal. Interestingly, AMP/MWA was higher in iNPH patients, suggesting a possible role of high ICP pulse pressure amplitudes in iNPH pathophysiology. Cerebrospinal reserve capacity and intracranial compliance is impaired in both groups and the pressure-volume relationship might be shifted towards lower ICP values in iNPH. The physiological influence of age on ICP and AMP/MWA requires further research.

## Background

Idiopathic normal pressure hydrocephalus (iNPH) is part of the large group of neurodegenerative diseases affecting the aging population. It is characterised by a clinical triad of progressive gait impairment, decline in cognitive function and incontinence. The natural history of iNPH is generally unfavourable with worsening neurological symptoms and reduced survival [1, 2]. Treatment of iNPH is surgical insertion of a ventriculo-peritoneal shunt (VP-shunt), which can lead to improved symptoms in selected patients and makes iNPH one of the few potentially treatable forms of dementia.

The pathophysiological processes of iNPH remain incompletely understood, resulting in a lack of clear diagnostic criteria. This makes selection of patients for surgical treatment and differentiation from other neurodegenerative diseases a challenge. Current evidence indicates that iNPH is thus underdiagnosed and undertreated [3]. It is now well accepted that iNPH is not only an imbalance of cerebrospinal fluid (CSF) production and reabsorption, but represents a more complex disturbance of CSF dynamics that becomes symptomatic at the late stages of life. There is therefore a need for better understanding of the pathophysiology of iNPH [4].

Late-onset idiopathic aqueductal stenosis of the adult (LIAS) is a less common form of chronic adult hydrocephalus [5]. The underlying pathology of LIAS is thought to be chronically impaired CSF flow through a narrowed aqueduct, typically leading to a well defined radiological entity with enlarged lateral and third ventricles (triventricular hydrocephalus), together with a convex tuber cinereum and lamina terminalis and dilatation of the third ventricular recesses, thought to arise from a chronic CSF accumulation and intracranial pressure (ICP) elevation “upstream” from the aqueduct. The aqueductal pathology is likely present for decades, possibly since childhood, and patients become symptomatic after years of compensated ventriculomegaly. The clinical presentation is very similar to iNPH with gait impairment, cognitive decline and incontinence, but the age at presentation is mostly younger. Headaches can also be part of the clinical presentation and are thought to arise from the chronic supratentorial ICP elevations.

The aim of this study was to investigate differences between the physiologic ICP waveform characteristics of patients with iNPH and LIAS. Despite the similar clinical features of these two forms of chronic adult hydrocephalus, we hypothesised that the relatively well-defined entity of LIAS would present with a distinct pattern of ICP features attributable to the impaired aqueductal CSF flow. Differentiation of the LIAS ICP-profile from the ICP-profile of iNPH might help to elucidate ICP characteristics for iNPH and LIAS and thus provide insight into pathophysiological processes of both diseases.

## Methods

We included patients treated at the Department of Neurosurgery, Wollongong Hospital, Illawarra Shoalhaven Local Health District, NSW, Australia between 2013–2020 and the Department of Neurosurgery, Eberhard Karls University Hospital, Tübingen, Germany, between 2009–2018 under the care of the two senior authors. Included patients underwent diagnostic intracranial pressure monitoring for investigation of iNPH or LIAS and were retrospectively identified from the ICP monitoring databases at both institutions. Clinical records, MRI and CT scans of the brain were reviewed for all patients.

### Patient characteristics, definition of iNPH and LIAS

**Fig. 1:**
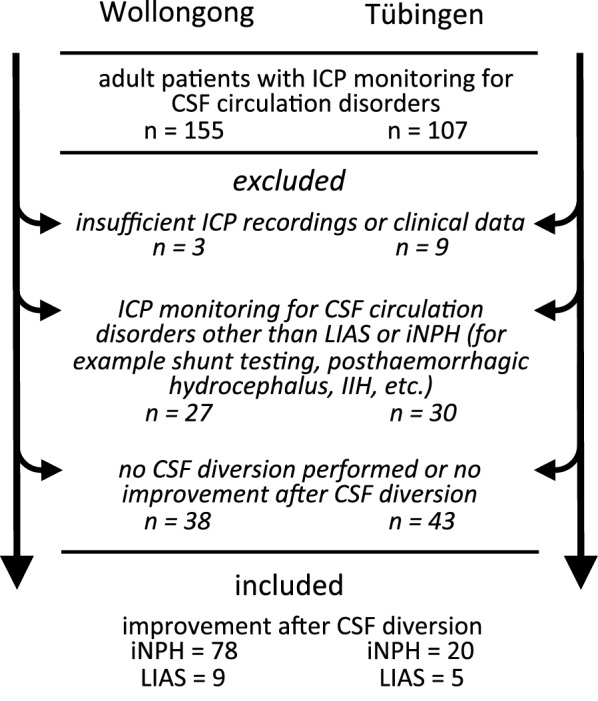
Selection process for the study population. Adult patient were identified from the ICP monitoring database at both institutions

The patient selection process and inclusion procedure is described in detail in Fig. 1. Patients with iNPH had probable or possible iNPH as described in the iNPH-guidelines [6], meaning they had ventricular enlargement on MRI and CT brain imaging without evidence of aqueductal stenosis or other CSF flow obstruction, together with appropriate clinical findings and no history of other causes of secondary NPH, such as brain haemorrhage or meningitis.

**Fig. 2:**
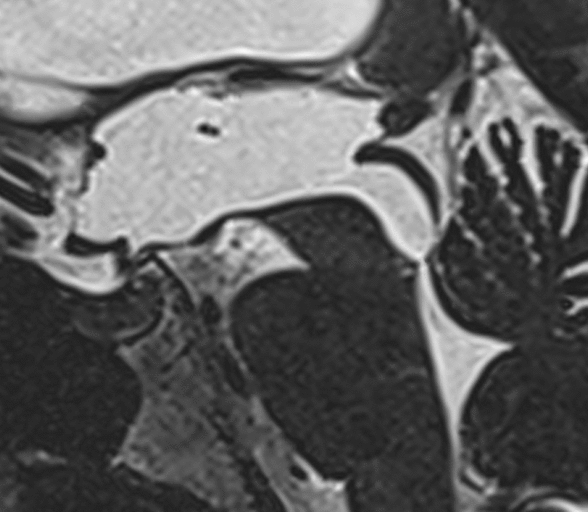
Sagittal T2 MRI image through the third ventricle, cerebral aqueduct and fourth ventricle showing typical findings for LIAS. Note the convex shape of the tuber cinereum and lamina terminalis, dilated third ventricle, stenosis of the distal aqueduct and normal sized fourth ventricle

LIAS patients had chronic symptoms as described by Fukuhara et al. with more than 6 months duration [5]. They had radiological evidence on MRI of aqueductal stenosis with a normal sized fourth ventricle, dilatation of the lateral and third ventricles with a convex tuber cinereum and lamina terminalis and dilatation of the third ventricular recesses, meaning downward bulging of the floor of the third ventricle and forward bulging of the lamina terminalis (Fig. 2). Patients with presentation of aqueductal stenosis with clinical signs and symptoms of elevated ICP were not part of this investigation as both study centres would typically not see the need for diagnostic ICP monitoring.

We only included patients with neurological improvement after CSF diversion in the final analysis. This is because we see improvement post shunting as the diagnostic gold standard and confirmation that patients suffered from symptomatic hydrocephalus and not other neurological disease. The disease severity of the iNPH patients was assigned retrospectively after review of the patients’ clinical notes using the Oslo-iNPH grading scale [7]. This simple scale allocates 1–5 points according to disease severity to each of the three domains of iNPH, gait impairment, cognitive impairment and incontinence. Patients receive a score of 3–15 points, with lower scores representing a more unfavourable clinical status. No specific scale to quantify disease severity exists for LIAS and we used simple symptom description for these patients. The indication for CSF diversion via VP-Shunt or endoscopic third ventriculostomy (ETV) was made by the treating teams after review of the individual clinical and radiological information, together with ICP data and CSF infusion studies. Outcome was assessed using the Oslo-iNPH scale from the clinical notes at the 1–3 month postoperative follow-up appointment. The Evans index to describe the degree of ventricular dilatation was recorded for each patient from diagnostic MRI or CT brain [8].

### ICP monitoring and data recording

Patients underwent diagnostic ICP monitoring for evaluation of chronic hydrocephalus as part of routine management. An ICP transducer was inserted through a right frontal burr hole. At Wollongong Hospital a Codman microsensor ICP transducer (Codman & Shurtleff, Raynham, MA, USA) was used, in Tübingen a Neurovent P sensor (Raumedic AG, Helmbrechts, Germany). ICP monitoring was performed on a neurosurgical ward for 1–3 nights. The ICP raw data signal was digitally sampled using ICM + software (Cambridge University, Cambridge, UK) [9]. During the study period the raw data sampling frequency increased from 50 to 200 Hz with 15 patients recorded at 50 Hz, 20 patients at 100 Hz and 77 patients at 200 Hz. To minimise artefacts in the ICP signal only overnight data during the patients’ sleep from a 10 h period from about 21:00 to 07:00 was analysed, typically from the first night a full 10 h recording was available. Artefacts, such as temporary disconnection of the ICP monitor, were manually eliminated after visual inspection of the ICP raw data.

### ICP waveform parameters

The ICP raw data was analysed using ICM + software version 8.3 for the following mean parameters from the 10-hour overnight monitoring:

1. The mean ICP was calculated.
2. The ICP pulse pressure amplitude (AMP): AMP was calculated from the amplitude of the fundamental harmonic in the frequency range of 30–140 beats per min (bpm) over 10 s intervals and averaged every 60 s.
3. Index of cerebrospinal compensatory reserve capacity (RAP): This is a marker of cerebrospinal compensatory reserve capacity and indirectly of compliance [10, 11]. It is calculated as the moving Pearson correlation coefficient between 30 consecutive samples of ICP and AMP averaged over 10 s. This 5 min time window was updated every 60 s. RAP just above zero indicates normal reserve capacity and compliance when the physiological slow wave fluctuations in ICP do not induce significant changes in AMP. RAP > 0.6 suggests impaired compliance when AMP correlates with slow wave ICP oscillations.
4. Heart rate (HR): HR was calculated from the frequency of the fundamental harmonic in the frequency range of 30–140 bpm over 10 s intervals and averaged every 60 s.
5. The ICP amplitude of the respiration induced waves (RESP): RESP was calculated from the amplitude of the fundamental harmonic in the frequency range of 7–30 bpm over a 120 s time window and updated every 60 s.
6. Respiratory rate (RR): RR was calculated from the frequency of the fundamental harmonic in the frequency range of 7–30 bpm over a 120 s time window and updated every 60 s.
7. The amplitude of the slow vasogenic waves of the ICP signal (SLOW): SLOW was calculated from the square root of the power in the frequency range of 0.3–3 bpm over a 10 min time window updated every 60 s [12].
  The ICP raw data was also analysed in the time domain using Sensometrics software (Sensometrics 4.0.2.4, dPCom A/S, Oslo, Norway) [13]. The following parameters were calculated:
8. The ICP mean pulse pressure wave amplitude in the time domain (MWA): The software algorithm identifies the amplitude of each single heartbeat induced pressure wave in the continuous ICP signal (the difference between diastolic minimum and systolic maximum pressure). MWA is calculated as the mean ICP pulse pressure wave amplitude for consecutive 6 s time windows. The software algorithm also identifies artefacts and time windows containing less than four valid heartbeat induced ICP waves are excluded.
9. Rise time of the ICP pulse pressure wave (RT): The RT was calculated as the time from the diastolic minimum to the systolic maximum for each heartbeat related ICP wave for consecutive 6 s time windows.
10. Rise time coefficient of the ICP pulse pressure wave (RTc): The RTc was calculated as the ratio of the amplitude over the rise time of each heartbeat induced ICP wave for consecutive 6 s time windows.

### Statistical analysis

Prism software (Version 8.4.2, GraphPad LLC, San Diego, CA, USA) was used for statistical analysis. We used the Mann-Whitney-U test and chi-square test for comparison of variables between groups. Multiple linear regression models were used to analyse for potential confounders on the dependent variables. Correlation between selected parameters was calculated using non-parametric Spearman correlation coefficient. The probability of a type-1 error (α) of 5 % was accepted as being statistically significant.

## Results

98 patients with iNPH showing improvement after VP-shunt insertion were included in this study. The median preoperative Oslo-score was 11 (range 4–14) and the median postoperative Oslo-score was 13 (range 7–15). They showed a median of 2 points (range 1–8) of improvement after shunt surgery. 14 patients with LIAS showing neurological improvement after VP-Shunt (n = 9) or ETV (n = 5) were included. 10 patients had improvement in gait, 8 in cognitive function, 7 in incontinence, and 7 reported better headache control. 11 patients had improvement in more than one domain. LIAS patients were younger and had a higher Evans index. Demographic and radiological data is summarised in Table 1.

**Table 1:** Demographic and radiological details of iNPH and LIAS groups

Variable	iNPH	LIAS	p value
No. of patients	98	14	
Age (years)	76.6 ± 5.6	53.8 ± 14.4	< 0.001
Female/male	33/65	8/6	0.09
Evans-index	0.39 ± 0.04	0.41 ± 0.05	0.03
Duration of artefact free ICP data obtained during 10 h of overnight monitoring (min)	593 ± 16	583 ± 39	0.67

Given values are the mean ± standard deviations or absolute numbers. p values from Mann–Whitney-U test and chi-square test

### ICP waveform characteristics

**Fig. 3:**
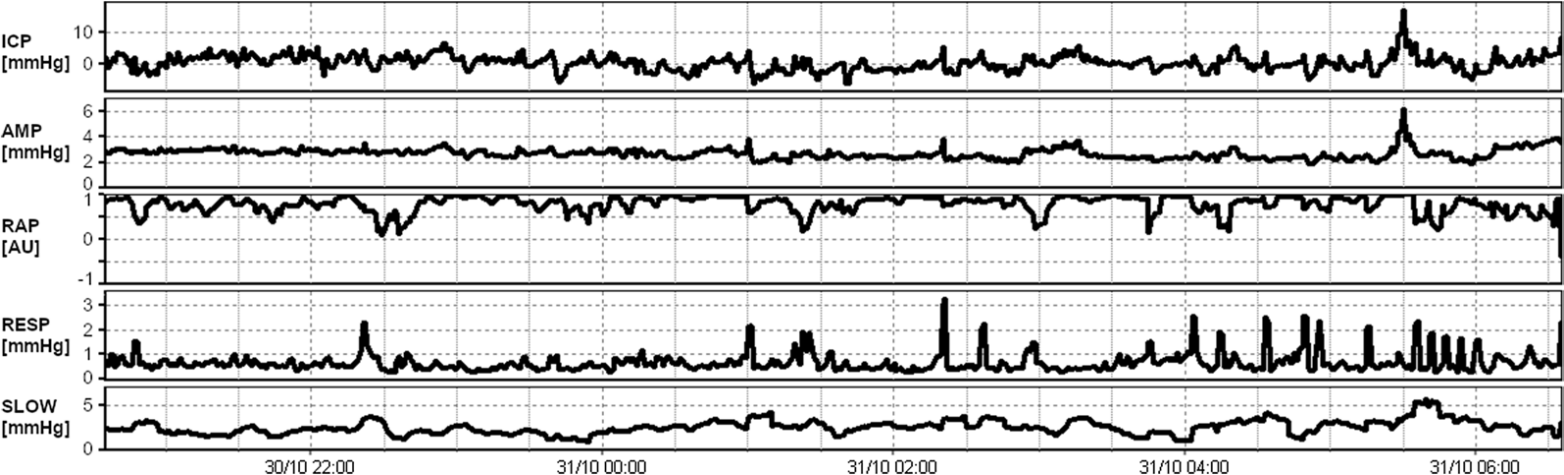
Typical 10 h overnight recording of ICP, AMP, RAP, RESP and SLOW

Figure 3 shows an individual example of 10 h overnight ICP monitoring and the waveform derived parameters AMP, RAP, RESP and SLOW.

**Fig. 4:**
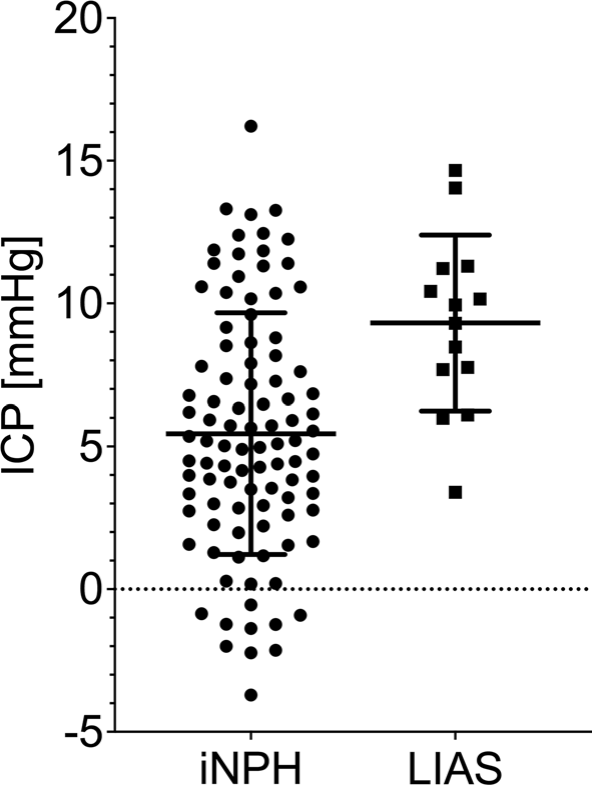
Values of ICP for iNPH and LIAS. Horizontal bars are the mean ± standard deviation. The difference between the groups was statistically significant (p = 0.001)

**Fig. 5:**
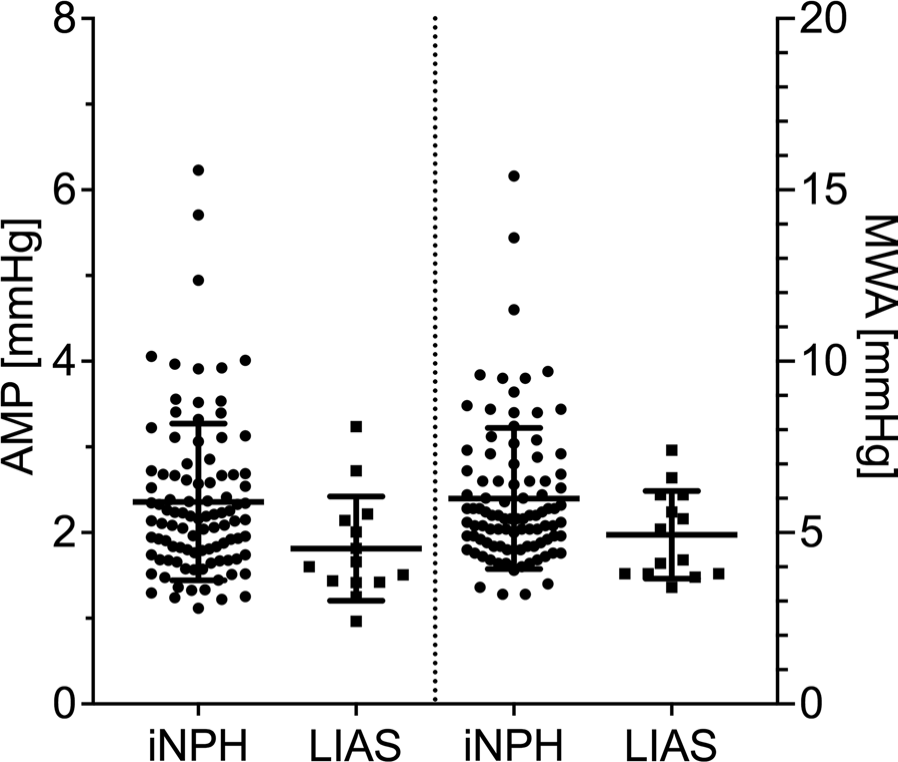
Values of AMP (left) and MWA (right) for iNPH and LIAS. Horizontal bars are the mean ± standard deviation. The difference between the groups was statistically significant (p = 0.012 for AMP, p = 0.049 for MWA)

**Fig. 6:**
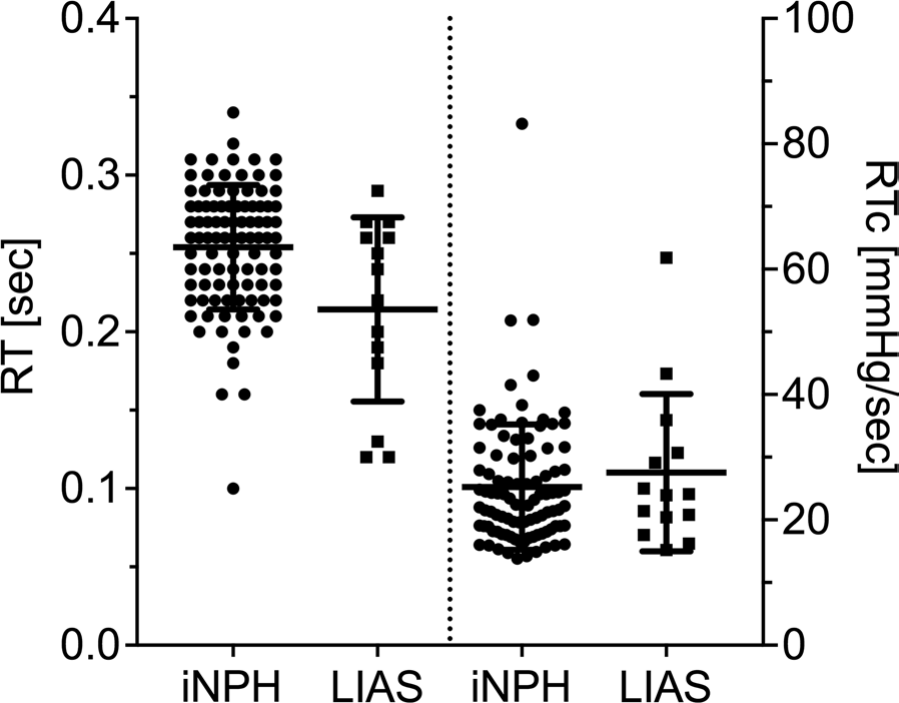
Values for RT (left) and RTc (right) for iNPH and LIAS. Horizontal bars are the mean ± standard deviation. The difference between the groups was statistically significant for RT (p = 0.014), but not for RTc (p = 0.61)

Table 2 shows the ICP waveform characteristics between the iNPH and LIAS groups. Mean ICP was higher in LIAS, but still in a range considered normal (Fig. 4). Mean AMP and MWA were higher in iNPH (Fig. 5). RT was higher in iNPH, but RTc was similar (Fig. 6). Of note, all these statistically different parameters displayed overlap in individual ICP and derived values between groups. Other ICP derived parameters were not statistically different between groups. Due to technical difficulties the ICP raw data from 2 iNPH patients from Tübingen, recorded at 50 Hz, could not be analysed with Sensometrics software and thus analysis of MWA, RT and RTc in the iNPH group is from 96 patients only.

**Table 2:** ICP derived parameters between iNPH and LIAS

Variable	iNPH	LIAS	p value
No. of patients	98	14	
ICP (mmHg)	5.4 ± 4.2	9.3 ± 3.0	0.001
AMP (mmHg)	2.36 ± 0.91	1.81 ± 0.59	0.012
MWA (mmHg)	6.0 ± 2.0	4.9 ± 1.2	0.049
RAP (AU)	0.67 ± 0.17	0.66 ± 0.11	0.59
HR (1/min)	69 ± 10	69 ± 11	0.88
RESP (mmHg)	0.52 ± 0.20	0.66 ± 0.59	0.69
RR (1/min)	14 ± 3	14 ± 2	0.64
SLOW (mmHg)	1.63 ± 0.49	1.78 ± 0.72	0.59
RT (s)	0.25 ± 0.04	0.21 ± 0.06	0.014
RTc (mmHg/s)	25.2 ± 9.9	27.5 ± 12.1	0.61

Given values are the mean ± standard deviations. p values from Mann–Whitney-U test

We found a significant correlation between ICP pulse wave amplitude calculated in the frequency domain (AMP) and time domain (MWA) of r = 0.93, p < 0.001.

In a multiple linear regression model age had a confounding effect on ICP between disease groups (iNPH versus LIAS) (p = 0.001, adjusted R^2^ = 0.15). In another multiple linear regression model age had no confounding effect on AMP (p = 0.19, adjusted R^2^ = 0.04). Both models were significant, but had a low adjusted R^2^.

### Differences between the two study centres

In patients with iNPH ICP was higher in Tübingen than Wollongong in the overnight monitoring (9.0 ± 3.8 mmHg versus 4.5 ± 3.8 mmHg, p < 0.001). RR was higher in Tübingen than in Wollongong (15.5 ± 2.9 bpm versus 13.9 ± 2.5 bpm, p = 0.024). AMP did not differ between centres in iNPH patients (2.1 ± 0.7 mmHg versus 2.4 ± 0.9 mmHg, p = 0.25, for Tübingen and Wollongong, respectively). MWA was also not different between centres in iNPH (5.6 ± 1.6 mmHg versus 6.1 ± 2.1 mmHg, p = 0.52, for Tübingen and Wollongong, respectively). Other clinical and monitoring parameters did not differ between centres in iNPH patients.

In LIAS patients the Evans index was lower in the Tübingen cohort (0.36 ± 0.05 versus 0.44 ± 0.02, p = 0.02). From the ICP derived parameters the following showed statistically different values between Tübingen and Wollongong, respectively: RR, 13.0 ± 1.1 bpm versus 15.1 ± 2.0 bpm, p = 0.04; RESP, 0.34 ± 0.07 mmHg versus 0.84 ± 0.68 mmHg, p = 0.012; and RTc, 19.7 ± 5.0 mmHg/s versus 31.9 ± 12.7 mmHg/s, p = 0.019). Other clinical and monitoring parameters did not differ between centres in LIAS patients.

## Discussion

This study investigated differences between the ICP profile of patients with iNPH and LIAS obtained from overnight recordings. We found mean ICP to be almost twice as high in LIAS patients compared to iNPH; however, mean ICP in LIAS was still in a range of ≤ 15 mmHg, generally considered normal. This is consistent with the idea that despite the often complex CSF pressure versus flow relationship in hydrocephalus the chronic compensated nature of the aqueductal flow impairment and supratentorial CSF accumulation causes a non-critical elevation of ICP and explains higher values compared to an iNPH cohort where aqueductal CSF flow is not obstructed and free communication between inner and outer CSF spaces can be assumed.

Interestingly, iNPH patients showed a significant elevation in AMP and MWA as compared to LIAS, despite the fact that they had a lower ICP. This is a most important finding, since with a given exponential pressure-volume relationship higher ICP are to be associated with higher AMP/MWA, and not vice versa. The increased AMP/MWA in iNPH at lower ICP indicates a very different pressure-volume relationship with the typical pressure-volume curve shifted to the left and a possibly narrower and steeper curve. This way the small intracranial volume changes caused by the cardiac pulsations induce larger ICP wave amplitudes at lower pressures.

Cerebrospinal reserve capacity and compliance measured with RAP were equally compromised in both groups, despite the higher average ICP values in LIAS. This again suggests a shift of the pressure-volume curve towards lower ICP values in iNPH, so that despite lower ICP a similar relationship between fluctuations in ICP and AMP results.

A previous investigation by González-Martínez et al. looked at differences between cerebrospinal fluid pressures, AMP and resistance to CSF outflow (R_out_) derived from the lumbar compartment during lumbar infusion studies in NPH patients with open or stenotic cerebral aqueducts [14]. In their investigation baseline ICP and AMP did not differ between groups, but R_out_ was higher in patients with an open aqueduct. This is again interesting as it indicates that the pressure differences between iNPH and LIAS patients obtained from the lumbar and cranial compartments might differ and should not be considered interchangeable.

Another possible explanation for the increased AMP/MWA in iNPH is diminished dampening of the cardiac induced pulsations in the intracranial compartment. Previous studies have reported increased MWA in iNPH, but the causes for these increased pulsations remain elusive at this stage [15]. They can theoretically lie anywhere between the heart, where they originate and the ICP sensor, where they are measured. Vascular stiffening, changes in the viscoelastic properties of the aging brain, impaired CSF absorptive capacity for arterial pulsations and other CSF specific factors all need to be taken into consideration [16, 17]. Whether the increase in AMP/MWA is a causative part of the pathophysiological disease process in iNPH or merely an associated epiphenomenon cannot be answered with this study. This study was also not designed to assess if increases in AMP/MWA can be used to select patients for VP-shunt surgery and may help differentiate shunt responders from non-responders. However, AMP/MWA certainly deserves further attention in iNPH research.

RAP was increased in both groups, indicating similarly impaired reserve capacity in both types of chronic hydrocephalus. SLOW, measuring vasogenic ICP slow wave or b-wave activity was also not different between groups. No threshold values for SLOW in chronic hydrocephalus exist, but the observed average values of above 1.5 mmHg can probably be considered abnormal [18].

The RT describes the time between the diastolic valley and systolic peak of the ICP pulse wave. It is thus, apart from intracranial compliance, dependant on the heart rate. RT was higher in iNPH, but the heart rate was similar between iNPH and LIAS. This might indicate that further analysis of the ICP pulse waveform morphology will be of interest in chronic hydrocephalus. This requires further sophisticated software analysis solutions, such as the MOCAIP algorithm, which are not available at our study centres [19].

This study has several limitations. ICP has been suggested to change with age and age has a confounding effect on our results. Older individuals likely have lower physiological ICP levels, but other factors such as vascular wall compliance are also changing with age. It is thus possible that the measured differences in ICP and AMP/MWA between the older iNPH and younger LIAS groups are mainly a function of age, and not disease. Our magnitude of ICP difference between iNPH and LIAS is however larger than a previous investigation measuring ICP in ‘pseudonormal’ patients across a wide range of age groups suggested. The data by Pedersen et al. found normal ICP to decrease by only 0.7 mmHg per decade of life [20]. Our statistical models to investigate the influence of age on ICP and AMP had low adjusted R^2^, likely due to the small number of LIAS patients, and thus need to be interpreted with caution. Other parameters describing CSF dynamics, such as resistance to CSF outflow, also change with age and further research to understand the physiological evolution of ICP and AMP/MWA with age is necessary [21].

The small group of LIAS patients prohibited a more definite statistical analysis. Chronic LIAS accounted for only approximately 5 % of all investigated adult CSF circulation disorders in both study centres. To increase the number of investigated LIAS patients to obtain a clearer statistical picture in this retrospective study was the main reason to combine data from two neurosurgical centres, both of which have regular experience with computerised ICP analysis of CSF circulation disorders using ICM + software. Whilst we were able to increase the number of analysed LIAS patients the combination of two centres might have also introduced bias into the analysis. For example, the proportion of iNPH and LIAS patients from both centres was different with a larger percentage of iNPH from Wollongong. In addition, ICP was higher in iNPH patients treated in Germany and lower in Australia. Whilst this might be incidental, it can also indicate bias in patient selection between hospitals or differences in clinical parameters that were not collected in this study, such as symptom duration or co-morbidities. Different ICP sensor equipment (Codman versus Raumedic) might have introduced further bias and should ideally be avoided in future prospective investigations.

## Conclusions

ICP is non-critically elevated in LIAS and lower in iNPH. AMP/MWA as a marker of intracranial heartbeat related pulsations is elevated in iNPH. The origin of these increased pulsations necessitates further research to understand this possible mechanism of iNPH better. In both disease groups intracranial reserve capacity is impaired with a possible shift of the pressure-volume curve towards lower ICP values in iNPH. The physiological influence of age on ICP and derived parameters requires additional investigations.

## Data Availability

The data that support the findings of this study are available upon reasonable request from the corresponding author [MJ]. The data are not publicly available due to them containing information that could compromise research participant privacy.

## Abbreviations

iNPH: Idiopathic normal pressure hydrocephalus
VP-Shunt: Ventriculo-peritoneal shunt
CSF: Cerebrospinal fluid
LIAS: Late-onset idiopathic aqueductal stenosis of the adult
ICP: Intracranial pressure
MRI: Magnetic resonance imaging
CT: Computed tomography
ETV: Endoscopic third ventriculostomy
AMP: Intracranial pulse pressure amplitude calculated in the frequency domain
bpm: Beats per minute
RAP: Correlation coefficient (R) between amplitude (A) and pressure (P), a marker of cerebrospinal compensatory reserve capacity
HR: Heart rate
RESP: ICP amplitude of the respiration induced waves
RR: Respiratory rates
SLOW: Amplitude of the slow vasogenic waves of ICP
MWA: Intracranial pulse pressure mean wave amplitude calculated in the time domain
RT: Rise time of the ICP pulse pressure wave
RTc: Rise time coefficient of the ICP pulse pressure wave
R_out_: Resistance to CSF outflow
